# A Mitochondrial-Related Gene Signature for Diagnosis and Immune Microenvironment Modulation in Lung Cancer and Venous Thromboembolism

**DOI:** 10.14740/wjon2815

**Published:** 2026-09-04

**Authors:** Xue Li Zhang, Ai Li Gao, Zhan Ju Liu

**Affiliations:** a Department of Respiratory and Critical Care Medicine, Beijing Shunyi Hospital, Beijing 101300, China

**Keywords:** Lung cancer, Venous thromboembolism, Mitochondria-related genes, Biomarkers, Diagnostic model, Immune infiltration

## Abstract

**Background:**

Lung cancer (LC) and venous thromboembolism (VTE) are closely associated, with VTE contributing to morbidity and mortality among patients with LC. We aimed to identify and characterize a mitochondrial-related transcriptomic signature shared between LC and VTE and to explore its association with immune microenvironment features.

**Methods:**

We applied a multiomics approach focused on mitochondrial-related signaling pathways. Publicly available transcriptomic datasets were analyzed using differential expression profiling and weighted gene co-expression network analysis to identify key regulatory genes. These genes were intersected with a mitochondrial gene set and subjected to functional enrichment analysis. Least absolute shrinkage and selection operator (LASSO) regression was used to identify candidate diagnostic genes validation. Immune cell infiltration was quantified, and associated regulatory mechanisms were explored.

**Results:**

Thirty-nine shared crosstalk genes were identified and were primarily enriched in mitochondrial metabolic processes. LASSO regression identified a five-gene candidate signature (*ACAA1*, *HSD17B10*, *MTIF2*, *THOP1*, and *PDE2A*). The model exhibited promising discriminatory performance (area under the curve > 0.9 in LC dataset and 0.7–0.9 in VTE dataset). These genes were significantly dysregulated and were associated with altered immune cell infiltration, particularly in dendritic cell and T cell subsets.

**Conclusion:**

We identified a mitochondrial-related gene signature reflecting shared transcriptomic correlates between LC and VTE. The signature showed variable performance across disease contexts and correlative associations with immune features, supporting its role as a candidate biomarker for further investigation. Prospective validation in independent clinical cohorts is required before any translational application.

## Introduction

Lung cancer (LC) is strongly associated with venous thromboembolism (VTE), a major cause of morbidity and mortality [1–3], encompassing both deep venous thromboembolism (DVT) and pulmonary embolism (PE). The complex bidirectional relationship between malignancy and thrombosis, often referred to as the “tumor–thrombosis axis,” is especially pronounced in LC. This increased risk is driven by multiple factors [4].

Although the clinical association between LC and VTE is well established, the molecular mechanisms underlying this comorbidity currently remain unclear, and there is a lack of sensitive and specific biomarkers for predicting thrombotic risk. The mitochondrion, traditionally regarded as the cellular energy center, is now recognized as a key regulator of both tumorigenesis and thrombogenesis. Beyond ATP production, mitochondria regulate apoptosis, reactive oxygen species (ROS) generation, calcium homeostasis, and metabolic reprogramming—processes frequently dysregulated in tumor and inflammatory environments that promote thrombosis [5]. Mitochondrial dysfunction may contribute to a prothrombotic state in malignancy by increasing tissue factor expression, promoting the release of procoagulant microparticles, and inducing platelet activation [6]. Therefore, mitochondria-related genes (MRGs) represent a promising but underexplored avenue for understanding the molecular interplay between LC and VTE.

Leveraging an integrative bioinformatics approach, we aimed to characterize the shared molecular architecture of LC and VTE [7]. We hypothesized that mitochondrial dysfunction serves as a central biological link between these conditions. Specifically, we sought to identify mitochondrial-related crosstalk genes (CGs) common to both diseases, characterize their associated biological pathways and immune infiltration patterns, and derive a candidate mitochondrial-related transcriptomic signature shared between LC and VTE. Rather than establishing a clinically validated diagnostic or predictive tool, this study treats the identified signature as a transcriptomic correlate that may inform future risk stratification and mitochondria-focused therapeutic exploration [8–11]. Our findings provide hypotheses for understanding the tumor–thrombosis axis and highlight the need for prospective cohort validation before any translational application.

## Methods

### Data retrieval and curation

Transcriptomic data were acquired from the Gene Expression Omnibus (GEO) repository via the GEOquery package in R [12, 13]. Four publicly available datasets were included: two LC datasets (GSE30219 [13] and GSE19188 [14]) and two VTE datasets (GSE48000 [10] and GSE19151 [15]). All samples were derived from *Homo sapiens*. Detailed characteristics of the LC and VTE datasets are provided in Supplementary Material 1 (wjon.elmerpub.com).

#### LC datasets

GSE30219 (training set): 293 lung tumor samples (LC group) and 14 adjacent nontumorous lung tissue samples (control group), profiled on the GPL570 platform (Affymetrix Human Genome U133 Plus 2.0 Array). Tissue source: lung. GSE19188 (validation set): 91 lung tumor samples and 65 normal lung tissue samples, profiled on the GPL570 platform. Tissue source: lung.

#### VTE datasets

GSE48000 (training set): 107 patients with VTE (stratified into high-, moderate-, and low-risk groups) and 25 healthy controls, profiled on the GPL10558 platform (Illumina HumanHT-12 v4.0 Expression BeadChip). Sample source: whole blood. GSE19151 (validation set): 70 confirmed VTE cases and 63 healthy controls, profiled on the GPL571 platform (Affymetrix Human Genome U133A 2.0 Array). Sample source: blood.

#### VTE etiology and subtype information

The public metadata for GSE48000 and GSE19151 provided limited granularity regarding VTE subtype (e.g., deep vein thrombosis vs. PE), provoking factors (e.g., surgery, immobilization, active cancer), and anticoagulation status at the time of sampling. Where available, risk stratification labels (high/moderate/low) were recorded. This heterogeneity in available clinical annotation is acknowledged as a limitation.

No formal sample size calculation was performed *a priori*, as this study utilized pre-existing, fixed-size public datasets. All available samples meeting inclusion criteria were included in the analysis. The outcome of interest was a binary disease status (case vs. control), and the time horizon was cross-sectional, reflecting diagnosis at the time of sample collection.

#### Mitochondrial-related gene (MRG) set construction

To construct a comprehensive set of MRGs, we applied a dual-source approach. First, the keyword “mitochondria” was used to query the GeneCards database [16] to identify protein-coding genes with a relevance score > 1.0, yielding 3,660 candidate genes. Second, MRGs were extracted from published literature using the same keyword criteria [17, 18], resulting in 3,840 genes. The two gene lists were merged, and duplicate entries were removed to generate the final MRG set, provided in Supplementary Material 2 (MRG_set worksheet) (wjon.elmerpub.com).

#### Data normalization

To ensure comparability across datasets, data normalization was performed using the limma package (version 3.58.1) in R [8]. For each dataset, raw expression values were background-corrected, quantile-normalized, and log2-transformed where appropriate. Following normalization, the resulting expression matrices contained no missing values; therefore, no imputation or removal of samples/genes due to missing data was required. For LC, GSE30219 was used as the training and internal testing dataset, and GSE19188 served as the independent validation cohort. For VTE, GSE48000 was used as the training dataset, and GSE19151 served as the external validation cohort.

### Identification and characterization of differentially expressed genes (DEGs)

Samples were stratified according to the design of each dataset. Specifically, samples from GSE30219 and GSE19188 were assigned to the LC and healthy control groups, whereas samples from GSE48000 and GSE19151 were assigned to the VTE and control groups, respectively. The identification of DEGs between disease and control cohorts was performed utilizing the limma package within the R environment. We applied specific selection criteria, defining DEGs as those exhibiting an absolute log2 fold change (|logFC|) exceeding 0.25 alongside a P-value below 0.05. Specifically, transcripts displaying a logFC greater than 0.25 with statistical significance (P < 0.05) were classified as upregulated, whereas those with logFC < −0.25 (P < 0.05) were considered downregulated. Volcano plots were generated using ggplot2 (version 3.4.4) to visualize overall expression patterns.

To identify robust LC-associated DEGs (LCDEGs), we selected genes that met the defined thresholds (|logFC| > 0.25, P < 0.05) in both GSE30219 and GSE19188. These overlapping genes were visualized using heatmaps generated with the pheatmap package (version 1.0.12 [19]). Similarly, VTE-associated DEGs (VTEDEGs) were identified by applying the same criteria to GSE48000 and GSE19151, and the overlapping genes were visualized using pheatmap.

### Weighted gene co-expression network analysis (WGCNA)

WGCNA represents a systems biology methodology designed to uncover candidate biomarkers and therapeutic targets through the assessment of gene co-expression architectures and their correlations with phenotypic characteristics [20]. All analyses were conducted using the WGCNA package in R [21]. Initially, pairwise correlation coefficients were computed across the entire gene set and subsequently raised to a soft-thresholding power to achieve a scale-free network topology. Following this, hierarchical clustering was employed to construct dendrograms, wherein distinct branches represented gene modules identified by unique color codes. Module significance was then assessed.

#### For the LC cohort (GSE30219)

The top 50% most variable genes were selected. Parameters: minimum module size = 200, soft-thresholding power = 5 (scale-free topology fit index > 0.85), module merging cut height = 0.1. Modules with |Pearson’s r| > 0.3 between module eigengene and phenotype (LC vs. control) were selected as the LC-associated WGCNA gene set (LC_WGCNA).

#### For the VTE cohort (GSE48000)

The top 60% most variable genes were selected. Parameters: soft-thresholding power = 16 (scale-free topology fit index > 0.80), minimum module size = 200, module merging cut height = 0.1. Modules with |r| > 0.3 were defined as the VTE-associated WGCNA gene set (VTE_WGCNA).

#### Identification of CGs

To identify shared transcriptomic correlates between LC and VTE, we first determined DEGs for each condition (LC_DEGs and VTE_DEGs). These gene sets were then intersected with the corresponding WGCNA module genes (LC_WGCNA and VTE_WGCNA) and the predefined MRG set. The final set of CGs was obtained from this multi-layered intersection and visualized using Venn diagrams.

### Functional annotation using Gene Ontology (GO) and Kyoto Encyclopedia of Genes and Genomes (KEGG) Pathway Enrichment

To elucidate the functional roles of the candidate genes, we performed GO enrichment profiling, which categorizes gene products across three distinct domains: biological processes (BPs), cellular components (CCs), and molecular functions (MFs) [22]. Concurrently, pathway-level enrichment was assessed by mapping these genes against the KEGG repository [23]. All analyses were performed using the clusterProfiler package in R [24]. No KEGG pathways reached statistical significance in this dataset.

### Gene set variation analysis (GSVA)

To assess pathway activation profiles at the single-sample resolution, we employed GSVA [11]. Curated gene sets from the Molecular Signatures Database [25] were applied to the LC dataset (GSE30219) to identify functional differences between tumor and control samples. Pathways with P < 0.05 were considered statistically significant. The same analytical approach was applied to the VTE dataset (GSE48000) to identify pathways distinguishing patients with VTE from healthy controls.

### Identification of key genes using least absolute shrinkage and selection operator (LASSO) regression

LASSO logistic regression was implemented using the glmnet package in R [25]. Predictor variables were internally standardized to zero mean and unit variance before model fitting, with coefficients transformed back to the original scale for the final model. The sample sizes between case and control groups in the training datasets (GSE30219 and GSE48000) showed some degree of imbalance. Given that LASSO logistic regression is relatively robust to moderate class imbalance, and to preserve the original data distribution for external validation, no resampling techniques or class weight adjustments were applied.

The initial candidate predictors were the DEGs identified in Section “Weighted gene co-expression network analysis (WGCNA)”. LASSO logistic regression was performed separately for LC (GSE30219) and VTE (GSE48000). The model was optimized by minimizing binomial deviance, and 10-fold cross-validation was used to determine the optimal penalty parameter (λ). The model corresponding to λ that minimized binomial deviance was selected. A fixed random seed (2022) [26] was set to ensure reproducibility of cross-validation splits.

### Development and validation of logistic regression models using signature genes

#### Model construction

To assess the relationship between the selected signature genes and disease status, we employed multivariable logistic regression. All identified signature genes were included as predictors, and individual regression coefficients were estimated. A composite risk score was then computed for each sample using the following formula:

$$\begin{array}{l} \text{Risk Score}=\sum_{i}{\text{Coefficient (gene}}_{\text{i}}\text{)}\\ \;\;\;\;\;*{\text{mRNA Expression (gene}}_{\text{i}}\text{)} \end{array}$$

A nomogram was constructed using the rms package (version 6.7-1) in R [27] to visualize the contribution of each gene to the predicted probability of disease. Calibration curves were generated to assess agreement between predicted probabilities and observed event rates. Decision curve analysis (DCA) was performed using the ggDCA package (version 1.1) [28] to evaluate the clinical net benefit of the model across threshold probabilities.

#### Validation strategy

##### 1) Internal validation

Receiver operating characteristic (ROC) curves were generated for the training datasets (GSE30219 for LC; GSE48000 for VTE) using the pROC package (version 1.18.5). The area under the curve (AUC) was computed to quantify discriminatory performance.

##### 2) Independent validation

The same risk score formula (with coefficients fixed from the training set) was applied to the independent validation datasets (GSE19188 for LC; GSE19151 for VTE). ROC analysis was repeated, and AUC values were reported.

##### 3) External validation

No fully independent external cohort (i.e., a completely separate dataset not used in either training or validation) with matched gene expression data and clinical phenotypes was available for this study. Therefore, external validation beyond the designated validation datasets was not performed and is acknowledged as a major limitation.

##### 4) Interpretation of AUC values

AUC values were interpreted as follows: 0.5–0.7 = low discriminatory accuracy; 0.7–0.9 = moderate accuracy; > 0.9 = high accuracy. The risk score was treated as a continuous measure reflecting the relative likelihood of disease versus control status. No pre-defined cut-off value was used to dichotomize the score into binary categories.

### Verification of differential expression and discriminatory utility for pivotal genes

To characterize the differential expression patterns of candidate genes, we compared their expression levels between disease and control groups using the same four datasets. ROC curves were constructed for each individual gene, and AUC values were computed using the pROC package. AUC interpretation followed the same thresholds as described in Section “Development and validation of logistic regression models using signature genes”.

### Assembly of protein–protein interaction (PPI) and regulatory networks

PPI networks constitute intricate systems of interconnected proteins that govern fundamental cellular activities, such as signal transduction, transcriptional control, energy metabolism, and cell cycle advancement. Investigating these interactions enables the elucidation of functional associations, signaling cascades, and molecular mechanisms linked to pathological conditions. To assemble a PPI network for the target genes, we used the STRING database [29]. The network was further expanded using GeneMANIA [30], which integrates genomic and proteomic data to identify genes with similar functions based on shared interaction patterns. GO [22] semantic similarity assessments were conducted utilizing the GOSemSim package within the R environment [31].

To examine transcriptional regulation, transcription factor (TF)–target interactions were retrieved from the ChIPBase database [32], and the regulatory interactions between TFs and messenger RNAs (mRNAs) were subsequently mapped and visualized utilizing the Cytoscape software platform. To investigate posttranscriptional regulation, microRNA (miRNA)–target interactions were retrieved from the StarBase v3.0 database [33].

To elucidate the intricate regulatory interplay among pivotal genes, we generated and rendered the resultant mRNA–miRNA network utilizing Cytoscape, thereby offering a holistic perspective on these molecular interactions.

### Characterization of immune infiltration landscapes associated with key genes using single-sample gene set enrichment analysis

ssGSEA [34] was performed to estimate the relative abundance of 28 immune cell populations in the LC dataset (GSE19188) and the VTE dataset (GSE19151). Enrichment scores were calculated for each sample. Group differences in immune cell scores were visualized using ggplot2. Spearman’s rank correlation was used to assess correlations among immune cell subsets and between gene expression and immune scores. Results were visualized as heatmaps (pheatmap) and bubble charts (ggplot2).

### Statistical analysis

All statistical analyses were performed in R (version 4.3.3). For two-group comparisons, the unpaired Student’s *t*-test was used for normally distributed data; otherwise, the Mann–Whitney U test (Wilcoxon rank-sum test) was applied. For comparisons involving three or more groups, the Kruskal–Wallis test was used. Correlations were assessed using Spearman’s rank correlation coefficient. All tests were two-sided, and P < 0.05 was considered statistically significant.

### Ethics approval and consent to participate

This study involved the analysis of publicly available, de-identified transcriptomic data from the GEO and TCGA databases. As no new human participants were recruited, and all data were anonymized, ethical approval and informed consent were not required for this study. The use of these data complies with the respective database’s access policies and ethical guidelines.

## Results

### Technology roadmap

**Figure 1:**
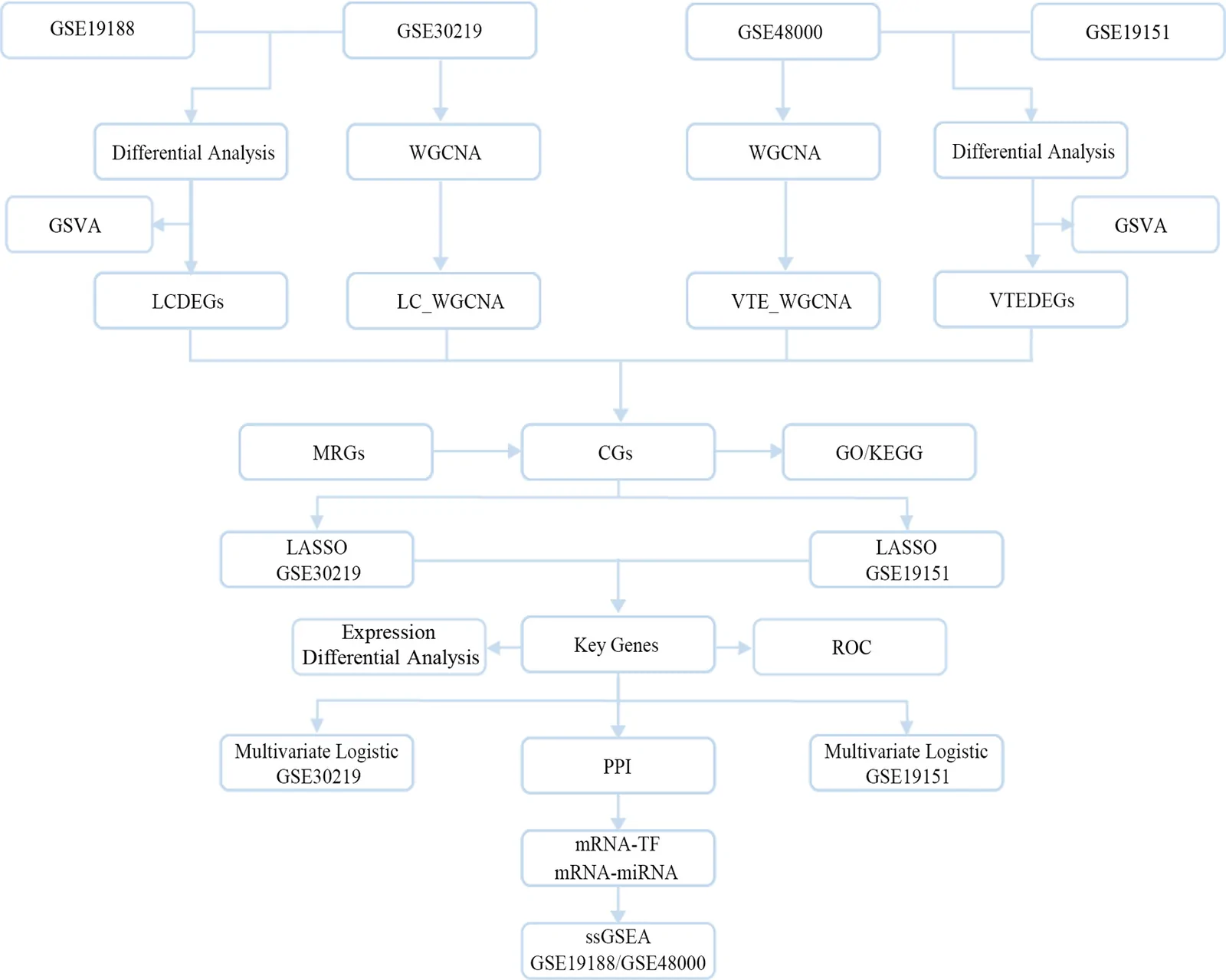
Technology roadmap. LCDEGs: lung cancer-associated differentially expressed genes; VTEDEGs: venous thromboembolism–associated differentially expressed genes; CGs: crosstalk genes; WGCNA: weighted gene co-expression network analysis; GSVA: gene set variation analysis; GO: Gene Ontology; KEGG: Kyoto Encyclopedia of Genes and Genomes; LASSO: least absolute shrinkage and selection operator; TF: transcription factors; ssGSEA: single-sample gene set enrichment analysis.

The overall workflow of this study is illustrated in Figure 1.

### Dataset preprocessing and normalization

To initiate the analysis, R packages were used to correct batch effects and normalize the LC datasets (GSE30219 and GSE19188) and the VTE datasets (GSE48000 and GSE19151). Boxplots (Supplementary Material 3, wjon.elmerpub.com) were generated to assess expression distributions before and after normalization. The results show that normalization reduced interbatch variability and improved data homogeneity across datasets. All data preprocessing and normalization steps described above were applied uniformly to the entire dataset. A comparison of these procedures across different sociodemographic subgroups (e.g., by sex or ethnicity) was not feasible, as the required detailed metadata were not consistently available in the public datasets used in this study.

### Identification of DEGs

**Figure 2:**
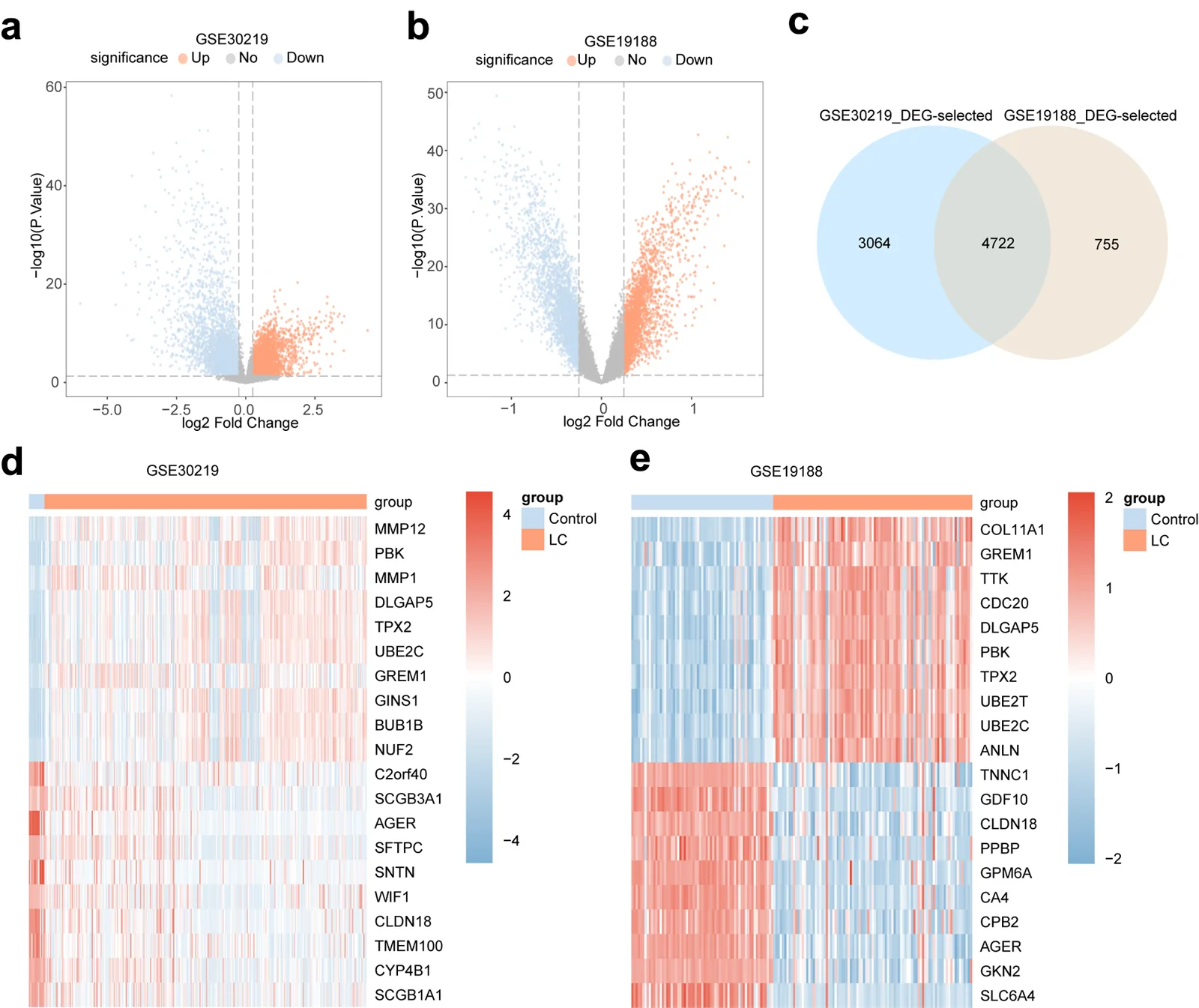
Differential gene expression analysis in LC. (a, b) Volcano plots of DEGs in GSE30219 (a) and GSE19188 (b), comparing LC samples with controls. (c) Venn diagram showing the overlap of DEGs between GSE30219 and GSE19188. (d, e) Heatmaps of the top 10 upregulated and top 10 downregulated LCDEGs in GSE30219 (d) and GSE19188 (e). In volcano plots, orange indicates LC samples, and light blue indicates controls. In heatmaps, red indicates higher expression and blue indicates lower expression. DEGs: differentially expressed genes; LC: lung cancer.

Differential expression analysis was performed using the limma package in R. In the LC datasets, 7,786 DEGs (4,167 up, 3,619 down) were identified in GSE30219 and 5,477 DEGs (2,518 up, 2,959 down) in GSE19188 (|logFC| > 0.25, P < 0.05) (Fig. 2a, b). Intersection yielded 4,722 common LCDEGs (Fig. 2c; Supplementary Material 2 (LC_DEGs and VTE_DEGs worksheets), wjon.elmerpub.com). In the VTE datasets, 7,257 DEGs (3,621 up, 3,636 down) were identified in GSE48000 (|logFC| > 0.25, P < 0.05) and 6,506 DEGs (2,825 up, 3,681 down) in GSE19151 (|logFC| > 0.25, P < 0.05) (Supplementary Material 4A, B, wjon.elmerpub.com). Intersection yielded 1,847 common VTEDEGs (Supplementary Material 4C, wjon.elmerpub.com; Supplementary Material 2 (LC_DEGs and VTE_DEGs worksheets), wjon.elmerpub.com). The top 10 up- and down-regulated DEGs for each condition were visualized by heatmaps (Fig. 2d, e, Supplementary Material 4D, E, wjon.elmerpub.com).

### WGCNA

**Figure 3:**
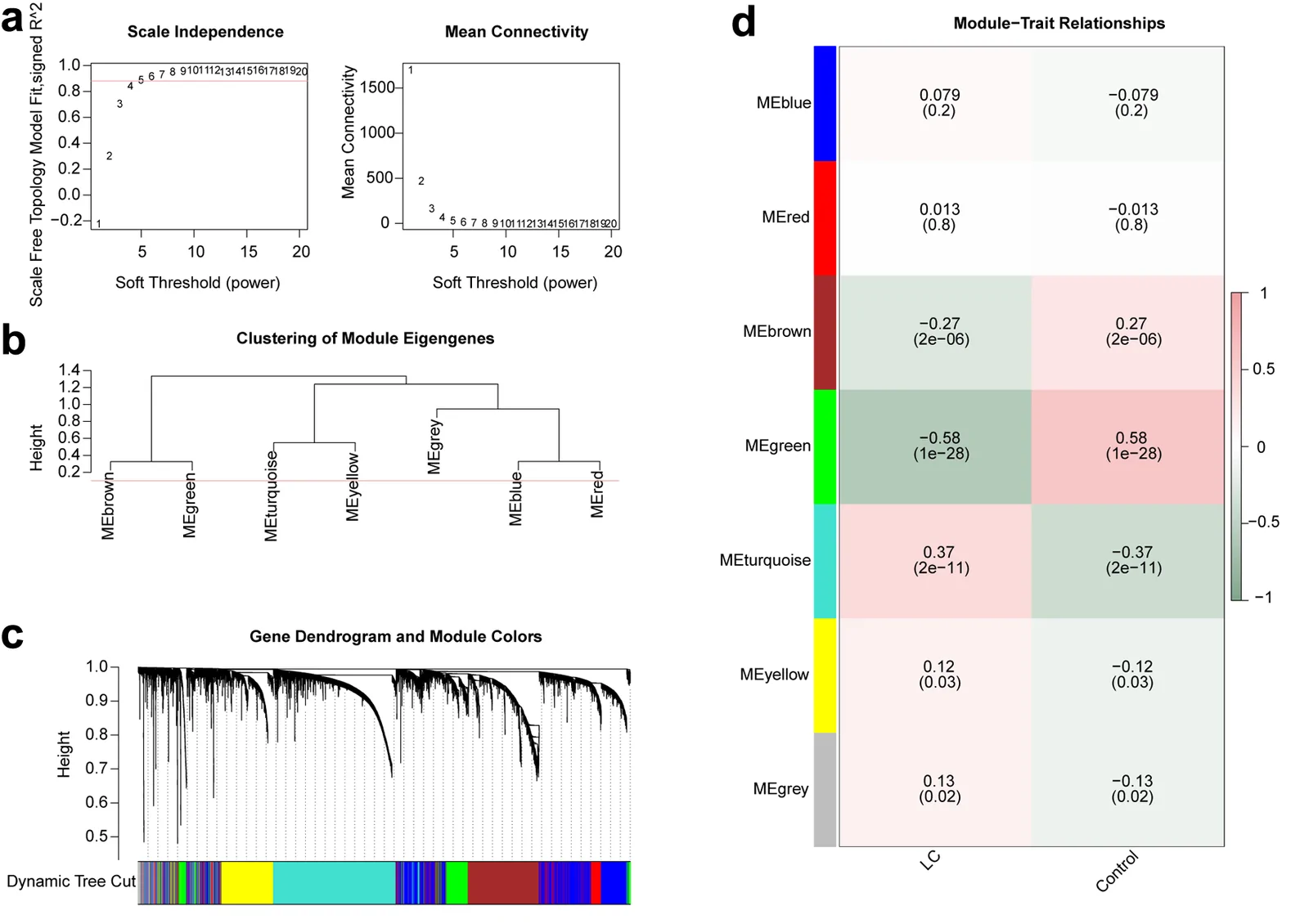
WGCNA of the GSE30219 dataset. (a) Determination of the optimal soft-thresholding parameter to establish a scale-free network architecture. The left panel illustrates the scale-free topology fit index across varying powers, while the right panel depicts mean connectivity. (b) Hierarchical clustering dendrogram of genes derived from the top 50% most variable genes. (c) Module assignment based on hierarchical clustering. The upper section illustrates the gene dendrogram, while the lower section depicts the associated gene modules using distinct color codes. (d) Heatmap of correlations between gene modules and sample groups (LC vs. controls). Correlation strength is indicated by |r| values, where 0.3–0.5 represents weak associations and 0.5–0.8 represents moderate associations. Color indicates direction of association: pink indicates positive correlations and gray-green represents negative correlations. WGCNA: weighted gene co-expression network analysis; LC: lung cancer.

WGCNA was performed to identify co-expression modules associated with disease status. The WGCNA-associated genes are provided in Supplementary Material 2 (LC_WGCNA and VTE_WGCNA worksheets) (wjon.elmerpub.com). For the LC dataset (GSE30219), the top 50% most variable transcripts were used with a soft-thresholding power of 5 (fit index = 0.88) and a module merging cut height of 0.1 (Fig. 3a). Seven modules were identified (Fig. 3b, c). Module–trait correlation analysis (Fig. 3d) identified MEgreen and MEturquoise as significantly correlated with LC status (|r| > 0.3), yielding 3,594 LC_WGCNA genes.

For the VTE dataset (GSE48000), the top 60% most variable genes were used with a soft-thresholding power of 16 (fit index = 0.80) (Supplementary Material 4F, wjon.elmerpub.com). Nine modules were identified (Supplementary Material 4G, H, wjon.elmerpub.com). Module–trait correlation analysis (Supplementary Material 4I, wjon.elmerpub.com) identified five modules (MEblue, MEblack, MEgreen, MEturquoise, MEgrey) meeting the |r| > 0.3 threshold, yielding 6,398 VTE_WGCNA genes.

To identify shared candidate genes, we intersected LCDEGs, VTEDEGs, LC_WGCNA, VTE_WGCNA, and the predefined MRG set. This intersection identified 39 CGs (Supplementary Material 4J, wjon.elmerpub.com; Supplementary Material 2 (Crosstalk_genes worksheet), wjon.elmerpub.com).

### GO enrichment analysis

To characterize the functional significance of the 39 CGs shared by LC and VTE, GO enrichment analysis was performed across the BP, CC, and MF domains. The full results are presented in Table 1.

**Table 1:** Results of GO Enrichment Analysis for CGs

ONTOLOGY	ID	Description	GeneRatio	BgRatio	P value	P.adjust	q value
BP	GO:0006753	Nucleoside phosphate metabolic process	6/37	495/18,800	0.00037551	0.04686129	0.03586955
BP	GO:1901293	Nucleoside phosphate biosynthetic process	5/37	257/18,800	0.00013997	0.04087129	0.03128456
BP	GO:0006163	Purine nucleotide metabolic process	5/37	394/18,800	0.00098912	0.05137613	0.0393254
BP	GO:0072521	Purine-containing compound metabolic process	5/37	416/18,800	0.00125965	0.05137613	0.0393254
BP	GO:0009117	Nucleotide metabolic process	5/37	487/18,800	0.00251283	0.06399664	0.04898565
CC	GO:0005759	Mitochondrial matrix	15/39	473/19,594	6.5367 × 10^−15^	8.1055 × 10^−13^	6.5367 × 10^−13^
CC	GO:0098798	Mitochondrial protein-containing complex	10/39	281/19,594	1.3819 × 10^−10^	8.5675 × 10^−9^	6.9093 × 10^−9^
CC	GO:0005743	Mitochondrial inner membrane	9/39	491/19,594	3.9314 × 10^−7^	1.625 × 10^−5^	1.3105 × 10^−5^
CC	GO:0000313	Organellar ribosome	4/39	81/19,594	1.9965 × 10^−5^	0.00045444	0.00036649
CC	GO:0005761	Mitochondrial ribosome	4/39	81/19,594	1.9965 × 10^−5^	0.00045444	0.00036649
MF	GO:0051087	Chaperone binding	3/39	106/18,410	0.00145835	0.06834594	0.04778702
MF	GO:0019001	Guanyl nucleotide binding	5/39	401/18,410	0.00149663	0.06834594	0.04778702
MF	GO:0032561	Guanyl ribonucleotide binding	5/39	401/18,410	0.00149663	0.06834594	0.04778702

GO: Gene Ontology; BP: biological process; CC: cellular component; MF: molecular function; KEGG: Kyoto Encyclopedia of Genes and Genomes; CGs: crosstalk genes.

**Figure 4:**
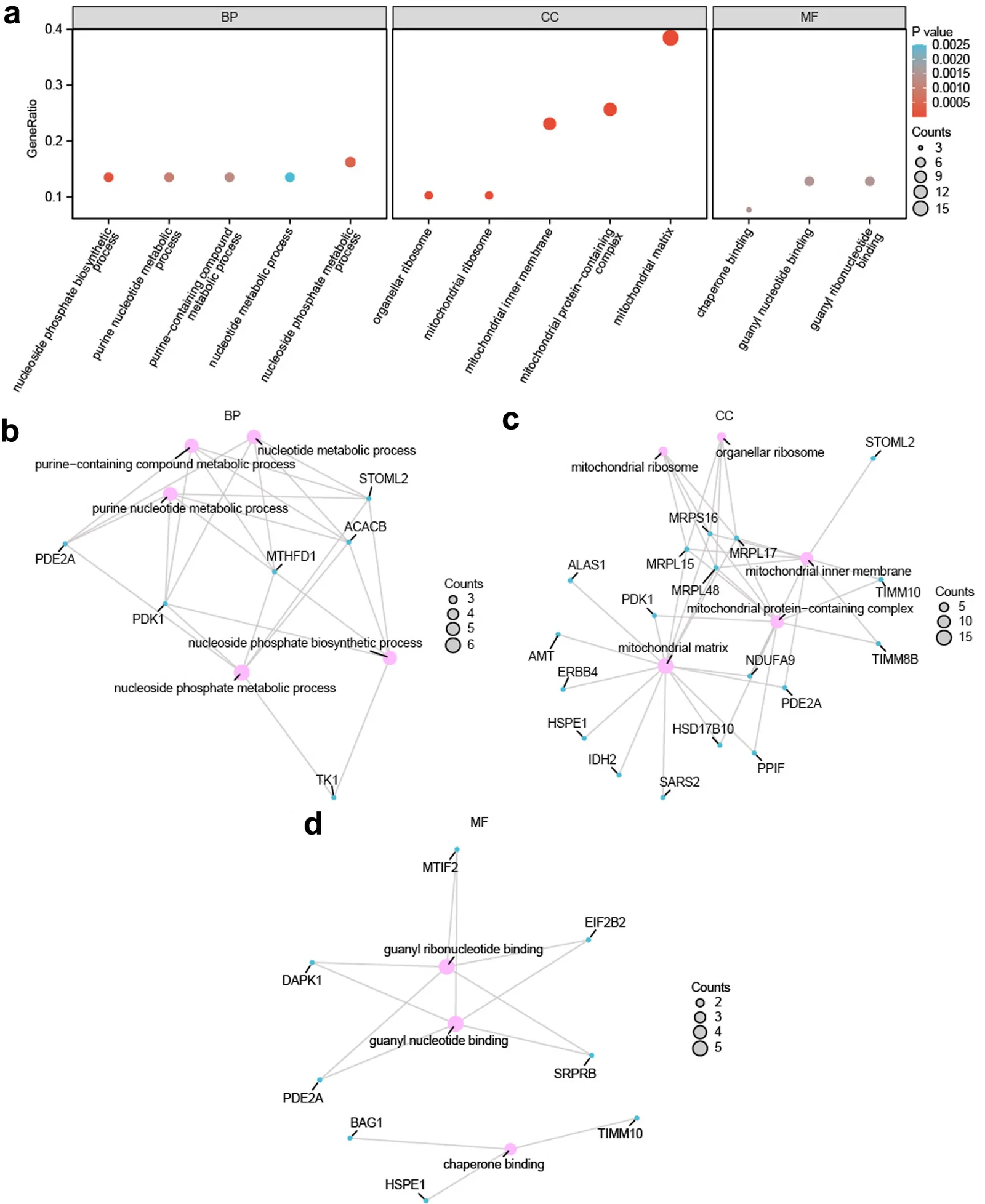
Gene Ontology enrichment analysis of crosstalk genes. (a) Bubble plot showing GO enrichment results for CGs across BP, CC, and MF. GO terms are shown on the x-axis. Bubble size indicates the number of genes, and color indicates statistical significance, with red indicating lower P-values and blue indicating higher P-values. (b–d) Network plots of enriched GO terms for CGs in BP (b), CC (c), and MF (d). Nodes represent GO terms (light red) and genes (light blue), and edges indicate their associations. Established enrichment thresholds were P < 0.05 and false discovery rate (FDR) < 0.25. CGs: crosstalk genes; GO: Gene Ontology; BP: biological process; CC: cellular component; MF: molecular function.

The CGs were primarily enriched in metabolic processes, particularly nucleoside phosphate metabolism. In the BP category, significant enrichment was observed in nucleoside phosphate biosynthesis, purine nucleotide metabolism, and pathways involving purine-containing compounds and nucleotide metabolism. In the CC category, enriched terms included the mitochondrial matrix, mitochondrial protein complexes, the inner mitochondrial membrane, and mitochondrial ribosomal structures. In the MF category, the genes were associated with chaperone binding and binding to guanyl nucleotides and guanyl ribonucleotides. These results are visualized using bubble plots in Figure 4a.

To further examine relationships among enriched terms, network plots were generated for BP, CC, and MF (Fig. 4b–d). In these networks, edges represent associations linking genes to GO terms, wherein the dimensions of each node correspond to the count of genes mapped to that specific term, highlighting the most prominent functional clusters.

### GSVA

**Figure 5:**
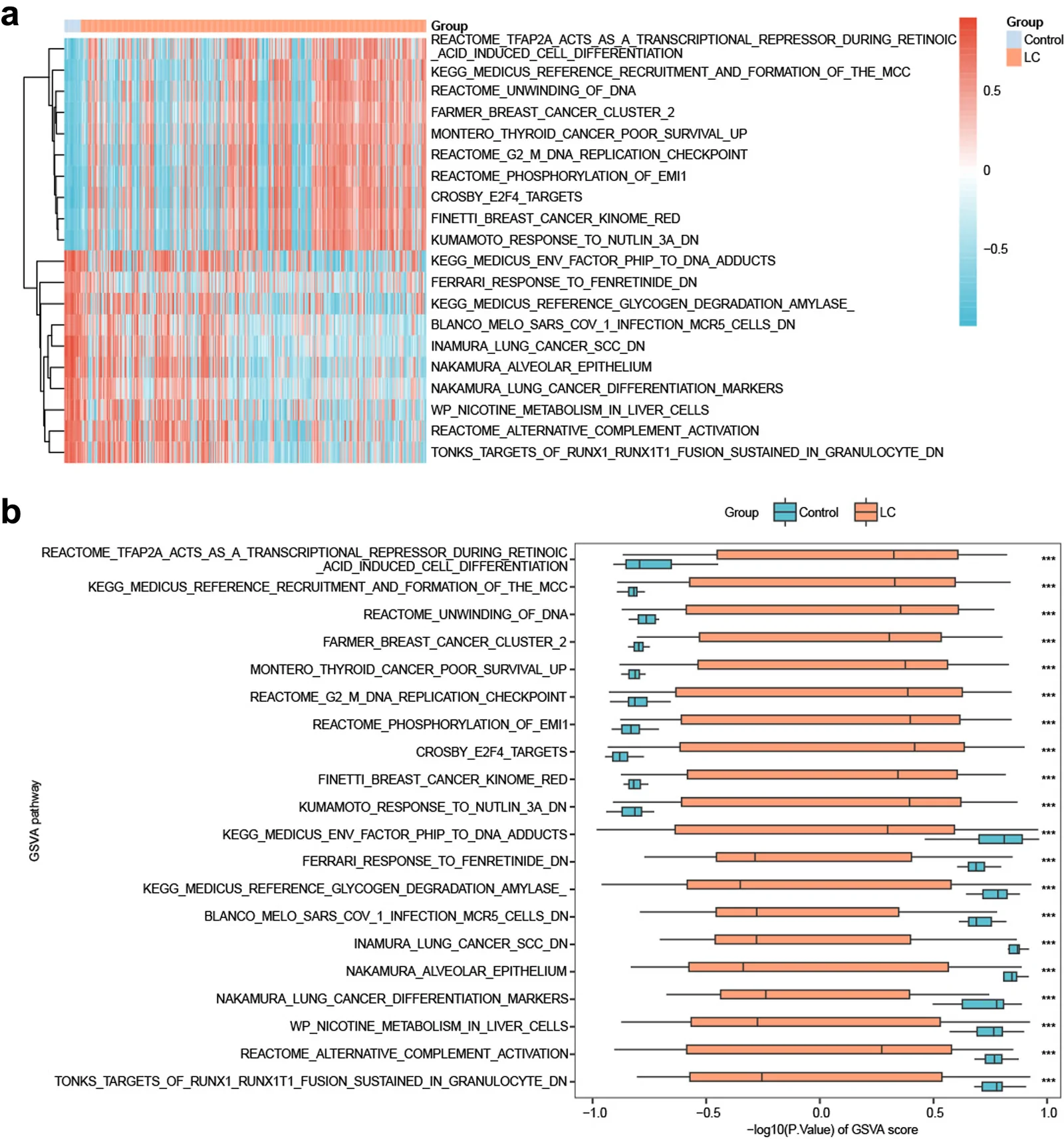
GSVA profiling of the GSE30219 LC dataset. (a) Heatmap of GSVA enrichment scores comparing LC samples with controls. Red corresponds to higher pathway activity; blue corresponds to lower activity. (b) Boxplots showing differences in pathway enrichment scores between the LC and control groups. Orange represents LC samples, and light blue represents controls. Pathway selection was conducted according to P < 0.05. ***P < 0.001, reflecting significant differences between groups.

To assess functional heterogeneity in the LC cohort, GSVA was performed on the GSE30219 dataset, which includes tumor and control samples, utilizing the c2.all.v2023.2.Hs.symbols.gmt repository of gene sets. Detailed GSVA results are provided in Table 2. The 20 most significantly enriched pathways (P < 0.05) were retained and ranked by the absolute value of logFC. Their enrichment patterns across LC and control groups were visualized using a hierarchical heatmap (Fig. 5a).

**Table 2:** Results of GSVA for GSE30219

	logFC	AveExpr	t	P value	Adjusted P value	B
CROSBY_E2F4_TARGETS	0.956108	0.049159	5.724314	2.421722828 × 10^−8^	6.513767243 × 10^−7^	8.781659
MONTERO_THYROID_CANCER_POOR_SURVIVAL_UP	0.89573	0.059486	5.865418	1.133022404 × 10^−8^	4.452922465 × 10^−7^	9.503221
REACTOME_PHOSPHORYLATION_OF_EMI1	0.884334	0.023819	5.451044	1.012865704 × 10^−7^	1.473852067 × 10^−6^	7.424824
FINETTI_BREAST_CANCER_KINOME_RED	0.883546	0.042484	5.746419	2.152025314 × 10^−8^	6.226890456 × 10^−7^	8.893763
KUMAMOTO_RESPONSE_TO_NUTLIN_3A_DN	0.878895	0.02374	5.305902	2.118769427 × 10^−7^	2.438604173 × 10^−6^	6.726374
REACTOME_G2_M_DNA_REPLICATION_CHECKPOINT	0.862235	0.022591	5.266889	2.576893984 × 10^−7^	2.800822227 × 10^−6^	6.541305
FARMER_BREAST_CANCER_CLUSTER_2	0.854006	0.029159	6.068145	3.713336651 × 10^−9^	2.403463232 × 10^−7^	10.56432
KEGG_MEDICUS_REFERENCE_RECRUITMENT_AND_FORMATION_OF_THE_MCC	0.850781	0.010201	5.496856	7.998335168 × 10^−8^	1.248156058 × 10^−6^	7.648502
REACTOME_UNWINDING_OF_DNA	0.844682	0.049118	5.458324	9.756630028 × 10^−8^	1.431236275 × 10^−6^	7.460265
REACTOME_TFAP2A_ACTS_AS_A_TRANSCRIPTIONAL_REPRESSOR_DURING_RETINOIC_ACID_INDUCED_CELL_DIFFERENTIATION	0.830487	0.050275	5.584729	5.063493258 × 10^−8^	9.553008036 × 10^−7^	8.081834
FERRARI_RESPONSE_TO_FENRETINIDE_DN	−0.74442	-0.02057	−5.93937	7.567324611 × 10^−9^	3.576921019 × 10^−7^	9.886972
WP_NICOTINE_METABOLISM_IN_LIVER_CELLS	−0.74873	0.015808	−5.02921	8.287434703 × 10^−7^	6.414700134 × 10^−6^	5.438605
TONKS_TARGETS_OF_RUNX1_RUNX1T1_FUSION_SUSTAINED_IN_GRANULOCYTE_DN	−0.76245	0.018083	−5.08997	6.172673057 × 10^−7^	5.239527177 × 10^−6^	5.716414
REACTOME_ALTERNATIVE_COMPLEMENT_ACTIVATION	−0.76295	0.038473	−4.83703	2.066088753 × 10^−6^	1.273823858 × 10^−5^	4.57858
NAKAMURA_LUNG_CANCER_DIFFERENTIATION_MARKERS	−0.76513	−0.00215	−6.52892	2.653051026 × 10^−10^	5.185639194 × 10^−8^	13.07993
KEGG_MEDICUS_REFERENCE_GLYCOGEN_DEGRADATION_AMYLASE	−0.77418	−0.00419	−4.94843	1.220863401 × 10^−6^	8.489696264 × 10^−6^	5.073616
NAKAMURA_ALVEOLAR_EPITHELIUM	−0.77422	−0.01295	−5.13427	4.970954288 × 10^−7^	4.522005209 × 10^−6^	5.920717
BLANCO_MELO_SARS_COV_1_INFECTION_MCR5_CELLS_DN	−0.77506	−0.03346	−6.34796	7.605661673 × 10^−10^	9.335810772 × 10^−8^	12.07507
KEGG_MEDICUS_ENV_FACTOR_PHIP_TO_DNA_ADDUCTS	−0.79025	0.027857	−4.70915	3.735469016 × 10^−6^	2.058810541 × 10^−5^	4.022264
INAMURA_LUNG_CANCER_SCC_DN	−0.89554	−0.01957	−7.03098	1.279898881 × 10^−11^	9.256228705 × 10^−9^	15.97746

GSVA: gene set variation analysis.

To validate these differences, the Mann–Whitney U test was used for group comparisons, and the results were presented as boxplots (Fig. 5b). GSVA identified multiple pathways exhibiting statistically significant disparities (P < 0.05) between LC and control groups, including *Crosby E2F4 Targets; Montero Thyroid Cancer Poor Survival Up; Phosphorylation Of EMI1; Finetti Breast Cancer Kinome Red; Kumamoto Response To Nutlin 3a Dn; G2 M DNA Replication Checkpoint; Farmer Breast Cancer Cluster 2; Medicus Reference Recruitment And Formation Of The MCC; Unwinding Of DNA; TFAP2A Acts as A Transcriptional Repressor During Retinoic Acid-Induced Cell Differentiation; Ferrari Response to Fenretinide Dn; Nicotine Metabolism in Liver Cells; Tonks Targets of RUNX1 RUNX1T1 Fusion Sustained in Granulocyte Dn; Alternative Complement Activation; Nakamura Lung Cancer Differentiation Markers; Medicus Reference Glycogen Degradation Amylase; Nakamura Alveolar Epithelium; Blanco Melo SARS-CoV-1 Infection MCR5 Cells Dn; Medicus Env Factor PHIP To DNA Adduct*.

To investigate functional alterations in VTE, GSVA was performed on the GSE48000 dataset using the c2.all.v2023.2.Hs.symbols.gmt gene set collection. Analytical parameters are provided in Table 3. The top 20 significantly enriched pathways (P < 0.05) were chosen and ordered according to absolute logFC values. Enrichment patterns between the VTE and control groups were illustrated through a heatmap (Supplementary Material 5A, wjon.elmerpub.com). Group differences were further assessed using the Mann–Whitney U test, with results presented as boxplots (Supplementary Material 5B, wjon.elmerpub.com). Twenty pathways showed significant differences (P < 0.05) between VTE and control groups, including *TCOF1 Targets (Jones), GSI Sensitivity Down (Palomero), Cellular Proteostasis, Electron Transfer in Complex III, Arsenic Exposure and Electron Transfer in Complex IV, Electron Transfer in Complex IV, Transport and Synthesis of PAPS, Mutant HTT and Electron Transfer in Complex III, Dual Hijack Model of VIF in HIV Infection, Parkin Pathway, TERT Pathway, Nef Protein and Signal Transduction, Negative Feedback Regulation of MAPK Pathway, STAT3 Signaling Pathway, TCA Cycle* Coupled *with Nutrient Utilization and Ovarian Cancer Invasiveness, KSHV miRNA Targeting of MHC-I Antigen Presentation, Shigella IpaB/CD and Integrin Signaling Pathway, HTLV-1 p12 and JAK-STAT Signaling Pathway, Maturation of SARS-CoV-1 Spike Protein, and the Ion Channel Pathway.*

**Table 3:** Results of GSVA for GSE48000

	logFC	AveExpr	t	P value	Adjusted P value	B
JONES_TCOF1_TARGETS	0.68535	−0.00891	8.419092	4.002037931 × 10^−14^	1.275010499 × 10^−12^	21.61831
PALOMERO_GSI_SENSITIVITY_DN	0.679906	−0.01643	8.4676	3.039355649 × 10^−14^	1.017621299 × 10^−12^	21.88801
WP_CELLULAR_PROTEOSTASIS	0.672864	−0.0243	7.490106	7.009193031 × 10^−12^	1.224407826 × 10^−10^	16.56116
KEGG_MEDICUS_REFERENCE_ELECTRON_TRANSFER_IN_COMPLEX_III	0.672106	0.012797	8.495715	2.590760166 × 10^−14^	8.845641371 × 10^−13^	22.04454
KEGG_MEDICUS_ENV_FACTOR_ARSENIC_TO_ELECTRON_TRANSFER_IN_COMPLEX_IV	0.650742	−0.00338	8.939515	2.042651044 × 10^−15^	9.783081028 × 10^−14^	24.53563
KEGG_MEDICUS_REFERENCE_ELECTRON_TRANSFER_IN_COMPLEX_IV	0.639247	−0.00433	8.814891	4.183298253 × 10^−15^	1.758930986 × 10^−13^	23.83248
REACTOME_TRANSPORT_AND_SYNTHESIS_OF_PAPS	0.611806	0.003844	7.886766	7.926471046 × 10^−13^	1.780255857 × 10^−11^	18.69348
KEGG_MEDICUS_VARIANT_MUTATION_CAUSED_ABERRANT_HTT_TO_ELECTRON_TRANSFER_IN_COMPLEX_III	0.578126	−0.0059	7.870238	8.687263705 × 10^−13^	1.93311665 × 10^−11^	18.60377
WP_DUAL_HIJACK_MODEL_OF_VIF_IN_HIV_INFECTION	0.577045	−0.04179	7.677574	2.515284738 × 10^−12^	4.916361952 × 10^−11^	17.56344
BIOCARTA_PARKIN_PATHWAY	0.573573	−0.01302	8.156217	1.762596724 × 10^−13^	4.756380413 × 10^−12^	20.16565
BIOCARTA_TERT_PATHWAY	−0.655	−0.00327	−12.3702	3.189052496 × 10^−24^	3.843871276 × 10^−21^	44.44821
REACTOME_NEF_AND_SIGNAL_TRANSDUCTION	−0.65533	0.012557	−9.53555	6.415944202 × 10^−17^	5.39536145 × 10^−15^	27.93171
REACTOME_NEGATIVE_FEEDBACK_REGULATION_OF_MAPK_PATHWAY	−0.65594	0.037132	−9.14853	6.104456894 × 10^−16^	3.476175768 × 10^−14^	25.72064
BIOCARTA_STAT3_PATHWAY	−0.66819	0.001079	−10.7443	5.123695773 × 10^−20^	1.323377423 × 10^−17^	34.93634
WP_TCA_CYCLE_NUTRIENT_USE_AND_INVASIVENESS_OF_OVARIAN_CANCER	−0.67075	0.033244	−9.16782	5.458713298 × 10^−16^	3.235853653 × 10^−14^	25.83035
KEGG_MEDICUS_PATHOGEN_KSHV_MIR1_2_TO_ANTIGEN_PROCESSING_AND_PRESENTATION_BY_MHC_CLASS_I_MOLECULES	−0.67317	0.008885	−7.70174	2.202486247 × 10^−12^	4.400105122 × 10^−11^	17.69336
KEGG_MEDICUS_PATHOGEN_SHIGELLA_IPAB_C_D_TO_ITGA_B_TALIN_VINCULIN_SIGNALING_PATHWAY	−0.68842	−0.0191	−11.7101	1.627713840 × 10^−22^	9.809688743 × 10^−20^	40.58619
KEGG_MEDICUS_PATHOGEN_HTLV_1_P12_TO_JAK_STAT_SIGNALING_PATHWAY	−0.70677	0.002033	−10.8561	2.635139635 × 10^−20^	7.255917307 × 10^−18^	35.5895
REACTOME_MATURATION_OF_SARS_COV_1_SPIKE_PROTEIN	−0.71873	−0.01869	−9.42971	1.19034545 × 10^−16^	9.158062016 × 10^−15^	27.32503
BIOCARTA_ION_PATHWAY	−0.79573	−0.00069	−11.9636	3.592743199 × 10^−23^	2.886968758 × 10^−20^	42.07005

GSVA: gene set variation analysis.

### Selection of pivotal genes through LASSO regression analysis

**Figure 6:**
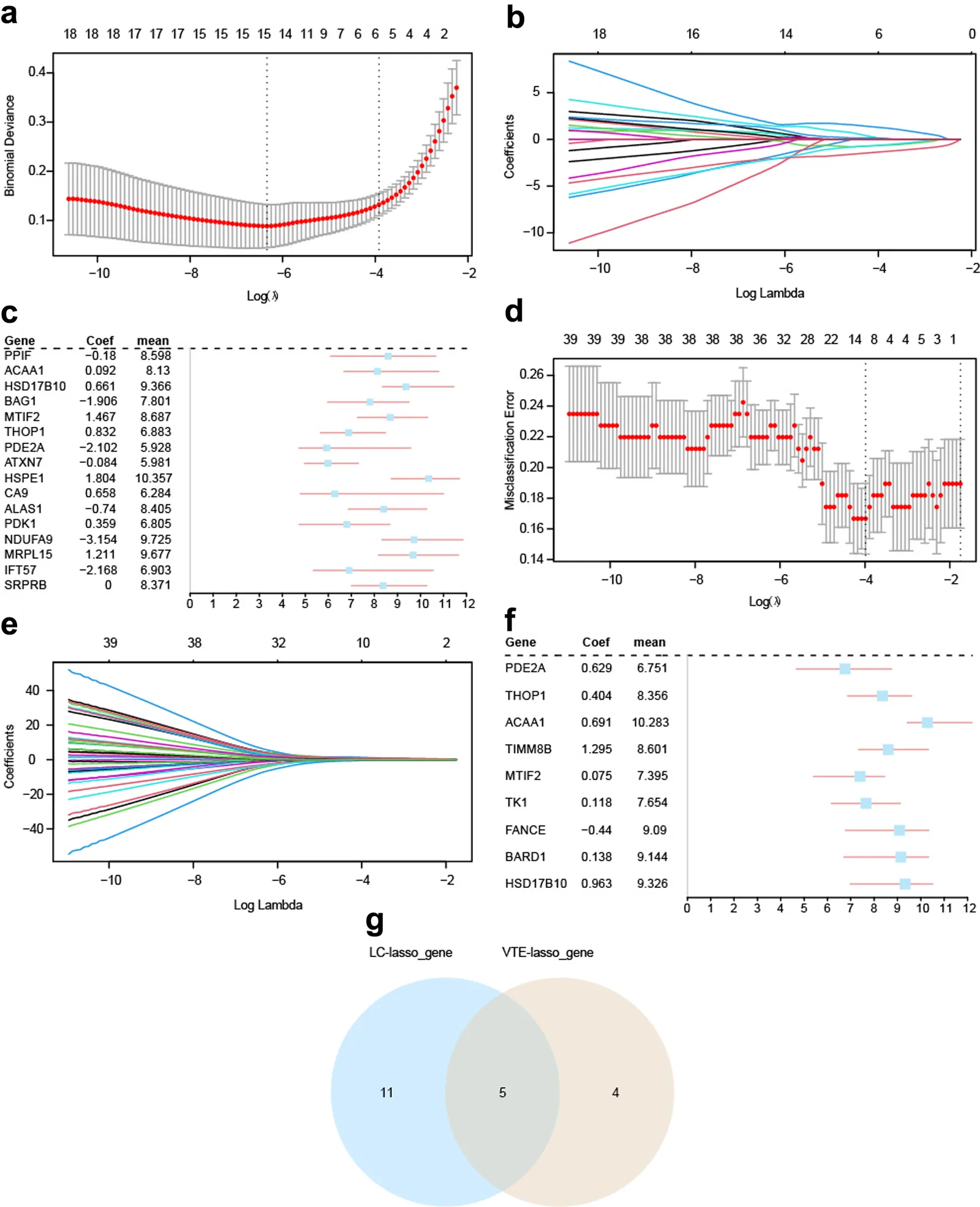
Identification of pivotal diagnostic genes. (a) LASSO cross-validation curve showing model performance in the LC dataset (GSE30219). (b) Coefficient paths illustrating variable selection in the LC dataset (GSE30219). (c) Forest plot showing effect estimates and confidence intervals of diagnostic genes associated with LC. (d) Diagnostic accuracy assessment of the CG-derived LASSO model applied to the VTE dataset (GSE48000). (e) Trajectory plots depicting the shrinkage paths of coefficients for predictors in the VTE cohort (GSE48000) under LASSO regularization. (f) Forest plot showing effect estimates and confidence intervals of diagnostic genes associated with VTE. (g) Venn diagram showing the overlap between LC- and VTE-associated diagnostic genes identified by LASSO. LC: lung cancer; VTE: venous thromboembolism; LASSO: least absolute shrinkage and selection operator; CGs: crosstalk genes.

To evaluate the discriminatory value of the 39 CGs in LC, LASSO regression was employed to establish a risk model. Model optimization and feature selection are illustrated by the cross-validation curve (Fig. 6a) and LASSO coefficient path (Fig. 6b). This analysis reduced the gene set to nine candidate genes associated with LC diagnosis: *PDE2A,* thimet oligopeptidase 1 (*THOP1*), acetyl-CoA acyltransferase 1 (*ACAA1*), *TIMM8B, MTIF2, TK1, FANCE, BARD1*, and hydroxysteroid 17-beta dehydrogenase 10 (*HSD17B10*). The corresponding odds ratios and 95% confidence intervals are presented in the forest plot (Fig. 6c). To evaluate diagnostic efficacy, a parallel LASSO regression analysis was performed to assess the diagnostic efficacy of the same 39 CGs in VTE. Model optimization and feature selection are illustrated by the cross-validation curve (Fig. 6d) and LASSO coefficient path (Fig. 6e). This analysis identified the same nine genes as key discriminatory features for VTE—*PDE2A, THOP1, ACAA1, TIMM8B, MTIF2, TK1, FANCE, BARD1*, and *HSD17B10*—with results summarized in the forest plot (Fig. 6f).

To identify shared biomarkers, a Venn diagram was constructed to compare gene signatures derived from the LC and VTE LASSO models (Fig. 6g). Five overlapping genes—*ACAA1, HSD17B10, MTIF2, THOP1*, and *PDE2A*—were identified as common biomarkers and were selected for subsequent analyses.

### Development and validation of logistic regression models using signature genes

To construct a discriminatory model for LC, we formulated a multivariable logistic regression model using five genes: *ACAA1, HSD17B10, MTIF2, THOP1*, and *PDE2A*. Regression coefficients were estimated for each gene, and a risk score was computed for every specimen within the training dataset (GSE30219) by combining gene expression values with their corresponding coefficients:

$$\begin{array}{l} \text{Risk Score}=-\text{0.660}\times ACAA1+\text{2.266}\times HSD17B10+\text{4.890}\times MTIF2\\ \;\;\;\;\;\;+\text{1.587}\times THOP1-\text{3.812}\times PDE2A\text{.} \end{array}$$

**Figure 7:**
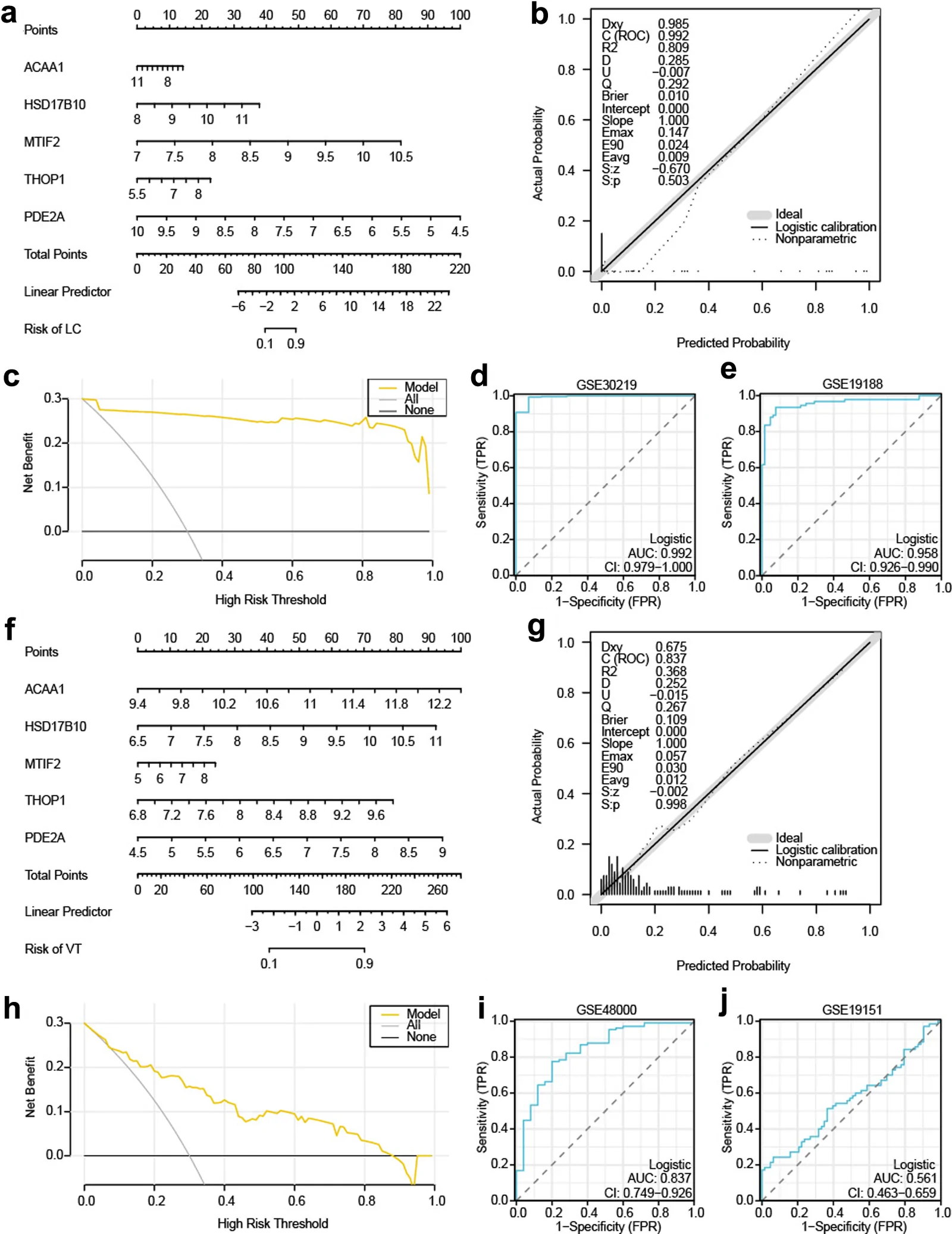
Development and validation of diagnostic logistic regression models using signature genes. (a) Nomogram for predicting LC based on the multivariable logistic regression model in GSE30219. (b) Calibration plot showing agreement between predicted and recorded findings in GSE30219. (c) DCA plot evaluating the practical application of the model in GSE30219 in clinical settings. (d, e) ROC curves showing model performance in the primary dataset GSE30219 (d) and the validation dataset GSE19188 (e). (f) Nomogram for predicting VTE utilizing a multivariable logistic regression framework in GSE48000. (g) Calibration plot showing consistency between anticipated and actual results in GSE48000. (h) DCA plot evaluating the practical application of the model in GSE48000. (i, j) ROC curves showing model performance in GSE48000 (i) and the validation dataset GSE19151 (j). In DCA plots, the y-axis quantifies net benefit, and the x-axis indicates threshold probability. In ROC curves, AUC values closer to 1.0 indicate superior diagnostic efficacy, values of 0.5–0.7 indicate suboptimal precision, and 0.7–0.9 indicate moderate precision. DCA: decision curve analysis; ROC: receiver operating characteristic; AUC: area under the curve; TPR: true positive rate; FPR: false positive rate.

The nomogram illustrated the relative contribution of each gene to the model (Fig. 7a). Calibration analysis (Fig. 7b) showed close alignment between predicted and observed values, confirming good model calibration. DCA on the training set (GSE30219) demonstrated superior net benefit compared to “treat-all” and “treat-none” strategies across varying threshold probabilities (Fig. 7c). ROC analysis showed high discriminatory performance in the training dataset (AUC = 0.992; Fig. 7d) and maintained strong performance in the validation dataset (AUC = 0.958; Fig. 7e).

To predict VTE, we constructed a multivariable logistic regression framework incorporating the same five genes (*ACAA1*, *HSD17B10*, *MTIF2*, *THOP1*, and *PDE2A*). Risk scores for samples in the training dataset (GSE48000) were calculated as follows:

$$\begin{array}{l} \text{Risk Score}=\text{1.773}\times ACAA1+\text{1.091}\times HSD17B10+\text{0.364}\times \text{MTIF2}\\ \;\;\;\;\;\;+\text{1.401}\times THOP1+\text{1.114}\times PDE2A\text{.} \end{array}$$

The nomogram illustrated the contribution of each gene to the VTE model (Fig. 7f). Calibration analysis (Fig. 7g) demonstrated that the model’s calibration curve closely approximated the ideal 45° reference line, confirming satisfactory predictive accuracy. DCA on the VTE training dataset (GSE48000) showed that the model provided a superior net benefit compared to “treat all” and “treat none” strategies within a threshold probability range of 0.1 to 0.8 (Fig. 7h). ROC analysis using the pROC package showed moderate discriminatory performance in the training dataset (GSE48000; AUC: 0.7–0.9; Fig. 7i) but lower performance in the validation dataset (GSE19151; AUC: 0.5–0.7; Fig. 7j). These findings indicate that model performance varies across VTE datasets and may be influenced by cohort-specific characteristics.

### Verification of differential expression patterns and discriminatory performance for individual genes

**Figure 8:**
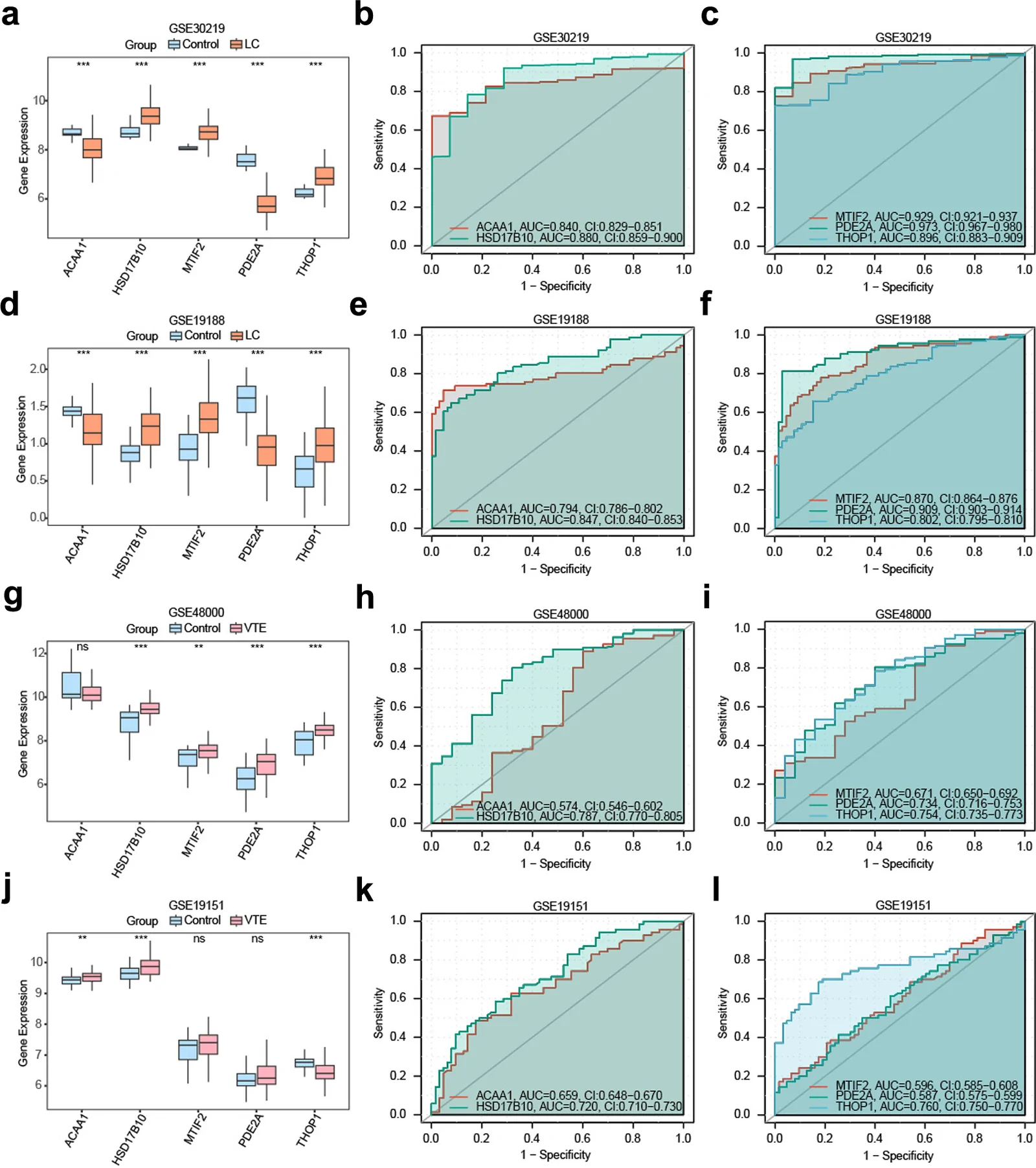
Validation of differential expression and diagnostic performance of candidate genes. (a) Expression comparison of key genes between LC and control samples in GSE30219. (b, c) Further analyses in GSE30219: (b) expression levels of *ACAA1* and HSD17B10; (c) ROC curves for *MTIF2*, THOP1, and PDE2A. (d) Expression comparison of key genes between LC and control samples in GSE19188. (e, f) Further analyses in GSE19188: (e) expression levels of ACAA1 and *HSD17B10*; (f) ROC curves for *MTIF2*, *THOP1*, and *PDE2A*. (g) Expression comparison of critical genes distinguishing VTE specimens from control cohorts in GSE48000. (h, i) Further analyses in GSE48000: (h) expression levels of *ACAA1* and *HSD17B10*; (i) ROC curves for *MTIF2*, *THOP1*, and *PDE2A*. (j) Expression comparison of key genes between VTE and control samples in GSE19151. (k, l) Further analyses in GSE19151: (k) expression levels of *ACAA1* and *HSD17B10*; (l) ROC curves for *MTIF2*, *THOP1*, and *PDE2A*. Statistical significance is denoted as follows: ns, not significant (P ≥ 0.05); **P < 0.01; ***P < 0.001. Diagnostic performance is interpreted based on AUC values: 0.5–0.7 reflects limited discriminatory power, 0.7–0.9 denote moderate performance, and > 0.9 indicates excellent accuracy. ROC: receiver operating characteristic; LC: lung cancer; AUC: area under the curve; VTE: venous thromboembolism. Color coding: blue, control; orange, LC; pink, VTE.

In GSE30219, all five genes were significantly dysregulated between LC and control samples (P < 0.001; Fig. 8a). ROC analysis showed that *MTIF2* and *PDE2A* achieved high discriminatory performance, whereas the remaining genes showed moderate performance (Fig. 8b, c). In GSE19188, all five genes remained significantly dysregulated (P < 0.001; Fig. 8d). *PDE2A* alone maintained an AUC > 0.9, whereas the remaining genes showed moderate performance (Fig. 8e, f).

In VTE, *HSD17B10*, *THOP1*, and *PDE2A* were significantly dysregulated in GSE48000 (P < 0.001; Fig. 8g), with moderate to strong AUC (0.7–0.9); *ACAA1* and *MTIF2* showed limited utility (AUC: 0.5–0.7) (Fig. 8h, i). In the independent validation (GSE19151), only *HSD17B10* and *THOP1* remained significantly dysregulated (P < 0.001; Fig. 8j), retaining moderate discriminatory performance (AUC: 0.7–0.9), while the remaining genes showed limited discriminative ability (AUC: 0.5–0.7) (Fig. 8k, l).

### Development of PPI and regulatory networks

**Figure 9:**
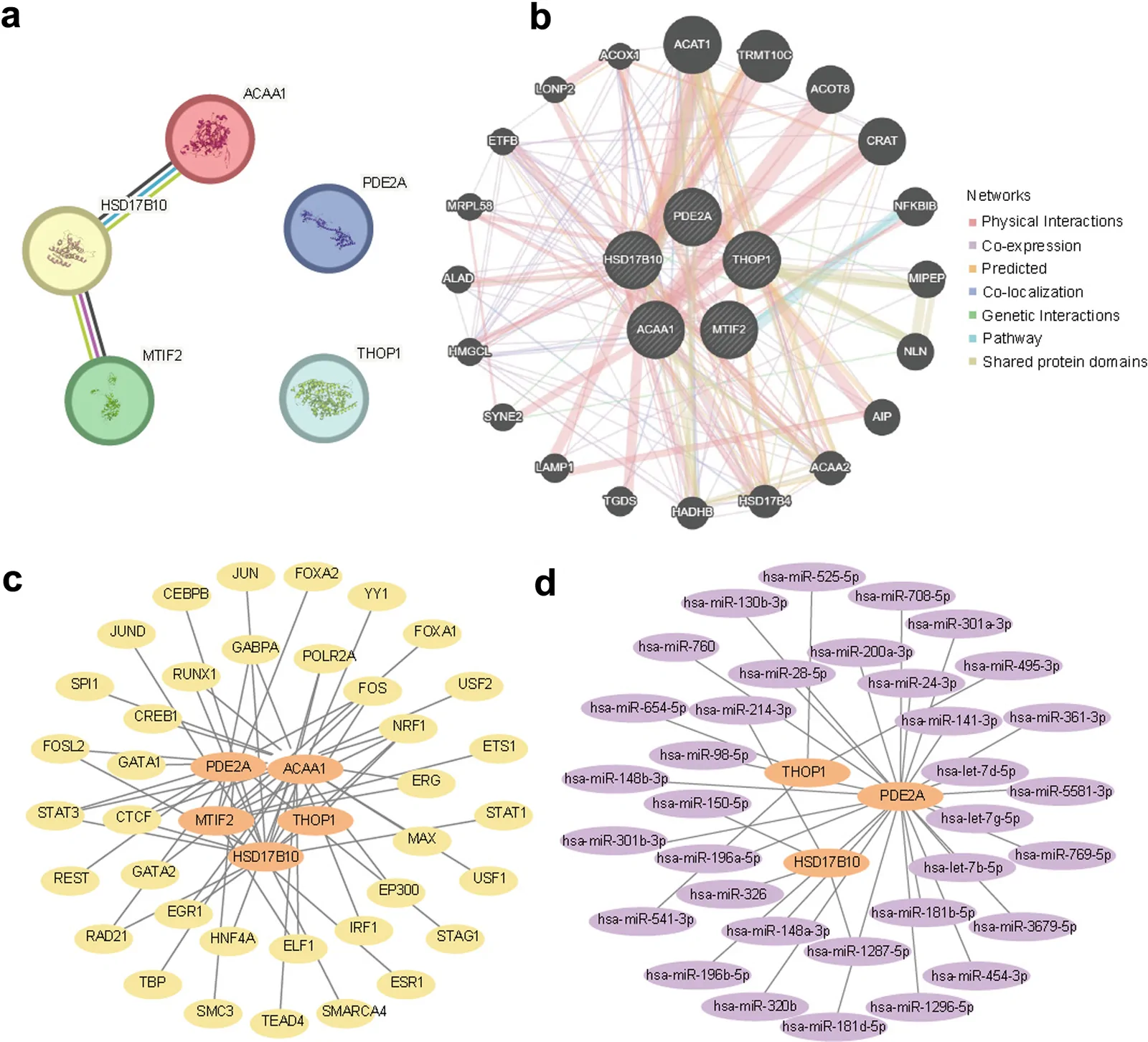
Investigation of regulatory networks governing key genes. (a) PPI network constructed using STRING, showing interactions among the five genes. (b) GeneMANIA network showing functional associations between the five genes and related proteins. Edge colors represent distinct interaction types. (c) mRNA–TF regulatory network showing interactions between genes and TFs. (d) mRNA–miRNA regulatory network showing interactions between genes and miRNAs. In panels c and d, orange nodes represent mRNAs, yellow nodes represent TFs, and purple nodes represent miRNAs. TF: transcription factor; PPI: protein–protein interaction.

To characterize the functional associations among the five genes, a PPI network was constructed using the STRING database (Fig. 9a). This analysis identified *ACAA1*, *HSD17B10*, and *MTIF2* as central nodes within the network. The interaction network was further expanded using GeneMANIA to include functionally related genes, generating an integrated network centered on the five target genes (Fig. 9b). Edge colors represent diverse types of associations, including co-expression and shared protein domains. The expanded network comprised the original five genes and 20 related proteins.

To examine transcriptional regulation, TF–target interactions were retrieved from the ChIPBase repository. A regulatory network linking the five signature genes and 37 TFs was constructed using Cytoscape (Fig. 9c), and the corresponding TF–mRNA interactions are provided in Supplementary Material 6 (TF_mRNA_interactions worksheet) (wjon.elmerpub.com). To assess posttranscriptional regulation, miRNA–target interactions were extracted from the StarBase repository. The miRNA–mRNA network linked three signature genes to 34 miRNAs (Fig. 9d), and the corresponding interactions are provided in Supplementary Material 6 (miRNA_mRNA_interactions worksheet) (wjon.elmerpub.com).

### ssGSEA-mediated profiling of immune infiltration patterns linked to signature genes

**Figure 10:**
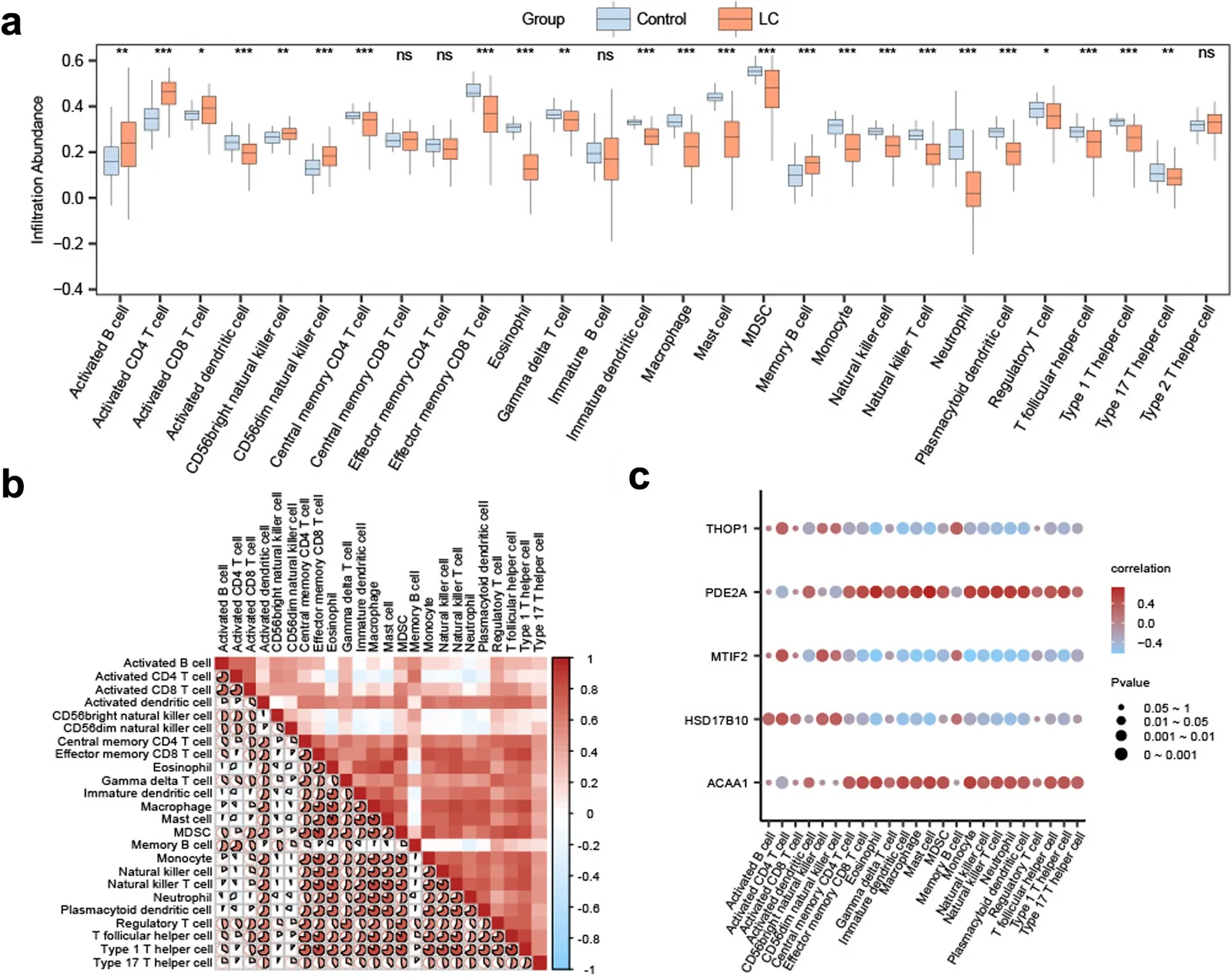
Immune infiltration profiling using ssGSEA in the GSE19188 dataset. (a) Boxplots comparing the abundance of immune cell subsets between control and LC samples. (b) Heatmap showing correlations among immune cell populations in GSE19188. (c) Bubble plot illustrating the correlations between gene expression levels and immune cell infiltration. Statistical significance is indicated as follows: ns, not significant (P ≥ 0.05); *P < 0.05; **P < 0.01; ***P < 0.001. Correlation strength is based on |r|, where < 0.3 reflects negligible, 0.3–0.5 weak, 0.5–0.8 moderate, and > 0.8 strong correlations. Color coding: light blue, control; orange, LC. In panels b and c, red indicates a positive correlation and blue indicates a negative correlation, with color intensity reflecting correlation strength. ssGSEA: single-sample gene set enrichment analysis; LC: lung cancer.

ssGSEA was performed to estimate the relative abundance of 28 immune cell populations in the LC dataset (GSE19188) and VTE dataset (GSE19151). In LC, 24 immune cell subsets showed statistically significant differences in inferred abundance between tumor and control groups (P < 0.05; Fig. 10a). In VTE, 21 immune cell subsets showed significant differences (P < 0.05; Supplementary Material 5C, wjon.elmerpub.com). Detailed cell type lists are provided in Fig. 10a and Supplementary Material 5C (wjon.elmerpub.com).

Correlation analysis revealed associations among immune cell populations. The strongest positive correlation was observed between eosinophils and mast cells in LC (Spearman’s r = 0.905, P < 0.05; Fig. 10b), and between activated CD4^+^ T cells and effector memory CD4^+^ T cells in VTE (r = 0.793, P < 0.05; Supplementary Material 5D, wjon.elmerpub.com).

Gene–immune correlation analysis showed that PDE2A had the strongest positive correlation with plasmacytoid dendritic cells in LC (r = 0.595, P < 0.05; Fig. 10c), whereas MTIF2 showed the strongest positive correlation with effector memory CD4^+^ T cells in VTE (r = 0.623, P < 0.05; Supplementary Material 5E, wjon.elmerpub.com). These correlations represent computational estimates of immune cell abundance and should be interpreted as associative rather than causal.

## Discussion

Our integrative analytical workflow, spanning multiomics data analysis and machine learning–based model construction, aligns with the growing use of computational approaches in oncology for biomarker discovery and mechanistic hypothesis generation [35]. Patients with LC have an increased risk of VTE, which contributes substantially to morbidity and mortality. Although clinical risk factors are well established, the shared molecular mechanisms linking these conditions remain unclear. In particular, the role of mitochondrial dysfunction—an important process in both tumor progression and thrombosis—has not been fully defined [1–6]. This gap limits the development of risk stratification strategies. To address this limitation, we performed an integrated multiomics analysis using publicly available transcriptomic datasets from LC and VTE cohorts to identify mitochondrial-related CGs and evaluate their functional and associative relevance.

This study identified a five-gene signature (*ACAA1*, *HSD17B10*, *MTIF2*, *THOP1*, *PDE2A*) consistently dysregulated in both LC and VTE, highlighting shared mitochondrial transcriptomic correlates underlying the tumor–thrombosis axis. The observed associations with immune infiltration further suggest potential links between these genes and immune microenvironment features.

Differential expression analysis identified 7,786 and 5,477 DEGs in LC datasets, and 7,257 and 6,506 DEGs in VTE datasets, reflecting extensive transcriptional reprogramming [1–3]. WGCNA identified key modules (MEgreen/MEturquoise in LC; five modules in VTE) significantly correlated with disease status (|r| > 0.3) [21]. Integration with mitochondrial gene sets yielded 39 CGs.

Functional enrichment revealed involvement of mitochondrial metabolic processes (nucleoside phosphate metabolism, mitochondrial matrix organization) [36–38], consistent with evidence that mitochondrial dysfunction—including oxidative stress, metabolic reprogramming, and DAMP release—contributes to both tumor progression and prothrombotic states [5, 6, 39]. Enrichment in chaperone binding further suggests disruption of mitochondrial protein folding and quality control, which may activate inflammatory and apoptotic pathways [40, 41].

GSVA showed enrichment of cell cycle pathways (e.g., G2/M DNA replication checkpoint) in LC and electron transport chain pathways in VTE [42, 43]. These findings suggest that impaired mitochondrial energy production may promote thrombotic susceptibility through ROS and altered calcium signaling [42, 44, 45], while mitochondrial metabolites support tumor proliferation in LC [46]. The concurrent dysregulation—bioenergetic disruption in VTE and increased anabolic demand in LC—highlights the central role of mitochondria in this comorbidity [47].

Among the five genes, *MTIF2* (mitochondrial translation) and *PDE2A* (cAMP signaling) may link mitochondrial stress to proinflammatory/procoagulant pathways [48, 49]. *ACAA1* (fatty acid oxidation) and *HSD17B10* (steroid metabolism) showed consistent performance across cohorts [50–53].

The signature demonstrated higher discriminatory performance in LC (AUC > 0.95) but variable performance in VTE (AUC 0.5–0.9). This discrepancy warrants careful interpretation. The stronger performance in LC datasets likely reflects the tumor-driven nature of the signature, as the feature selection process was predominantly informed by cancer transcriptomic structure. In contrast, the lower and more variable AUCs in VTE cohorts may be attributable to several factors. First, VTE is biologically heterogeneous, encompassing diverse clinical subtypes (deep vein thrombosis, PE, cancer-associated thrombosis, provoked and unprovoked events) that are often inadequately captured in public metadata. Second, differences in sample source (whole blood vs. buffy coat), processing protocols, and platform batch effects may introduce technical variability. Third, the signature may capture tumor-related inflammatory and mitochondrial programs more effectively than VTE-specific thrombotic processes. Prospective validation in well-characterized, harmonized cohorts with detailed VTE phenotyping is warranted to clarify these issues.

ssGSEA revealed extensive differences in inferred immune cell abundance in both conditions, affecting 24 immune cell types in LC and 21 in VTE (Supplementary Material 5C, wjon.elmerpub.com) [54, 55]. Consistent with recent reports on TME-regulating gene signatures in lung adenocarcinoma [56, 57], associations between mitochondrial genes and specific immune populations—including *PDE2A* with plasmacytoid dendritic cells and *MTIF2* with effector memory CD4^+^ T cells (Supplementary Material 5E, wjon.elmerpub.com)—suggest that these genes may influence disease progression through potential immune-related association [55, 58], extending the role of mitochondrial dysfunction to both immune dysregulation and thrombosis in the LC–VTE axis.

These findings align with the concept of immunothrombosis, wherein mitochondrial components released during cellular stress act as DAMPs, activating innate immune responses and promoting a prothrombotic state [50, 58, 59]. The association of *THOP1* with Tregs (Supplementary Material 5E, wjon.elmerpub.com) suggests a role for mitochondrial peptide processing in immune tolerance [60]. Supplementarily, mtDNA release may trigger NETosis, further linking mitochondrial dysfunction to thrombotic risk in cancer [59, 61]. Together, the enrichment of cell cycle pathways in LC and electron transport chain pathways in VTE supports a model in which mitochondrial dysfunction drives both tumor progression and thrombosis.

PPI network and mRNA–miRNA–TF regulatory analyses identified 37 TFs and 34 miRNAs, including inflammation-related NF-κB and STAT family members, suggesting potential avenues for future investigation linking inflammation, coagulation, and metabolic dysregulation [53, 54, 62].

The predicted miR-181–PDE2A regulatory axis links mitochondrial, immune, and thrombotic pathways to the shared pathogenesis of LC and VTE [55, 63]. PPI network analysis further indicated that *ACAA1* and *HSD17B10* may interact with pathways involved in inflammation, cell cycle regulation, and coagulation. These network interactions provide a gene-level framework connecting mitochondrial dysfunction in tumor cells to prothrombotic inflammatory responses in the vasculature [5, 64].

Future experimental studies could validate this five-gene signature in patient-derived liquid biopsies, such as plasma cfRNA or exosomal RNA, and in experimental thrombosis models. Such work may help determine whether the transcriptomic signal has direct relevance to thrombus formation or primarily reflects underlying tumor and host inflammatory states.

This study has several limitations. First, the diagnostic signature showed moderate performance in the external VTE validation dataset (GSE19151; AUC: 0.56–0.7), indicating limited generalizability. This variability, a common challenge in translational bioinformatics [7, 65], likely stems from clinical heterogeneity (e.g., comorbidities, VTE subtypes), differences in sample processing, and technical batch effects. Prospective validation in cohorts with matched tissue and blood samples from LC patients stratified by VTE status is required. Second, these computational findings require experimental verification through *in vitro* and *in vivo* studies specific to cancer-associated thrombosis. Third, single-cell and spatial transcriptomic approaches would help clarify the cellular context of these genes and may explain performance variability across datasets [66]. Fourth, evaluation at the protein level (e.g., expression, post-translational modifications, functional activity of *ACAA1* and *HSD17B10*) is necessary to confirm clinical relevance. Fifth, our study did not assess model performance heterogeneity across clinical centers or geographic regions, as detailed site-specific metadata were unavailable. Sixth, and importantly, this study did not include a fully independent external validation cohort with matched gene expression and clinical phenotypes; therefore, the generalizability of the signature remains to be established.

Overall, this study identifies a mitochondrial-related transcriptomic signature shared between LC and VTE, highlighting mitochondrial dysfunction as a potential shared feature linking oncogenesis and thrombosis. The identified gene signature and regulatory networks provide a foundation for future hypothesis-driven studies and require validation using prospective, multiomics, and experimental approaches.

### Conclusion

This integrative bioinformatics analysis identified an association between LC and VTE involving mitochondrial dysfunction and immune microenvironment dysregulation. We defined a five-gene mitochondrial-related signature (*ACAA1*, *HSD17B10*, *MTIF2*, *THOP1*, and *PDE2A*) that showed high discriminatory performance for LC and variable performance for VTE across validation datasets. These findings support the presence of a mitochondrial–immune–thrombosis axis in LC. The identified gene signature and associated immune infiltration patterns provide candidate biomarkers for future risk stratification, pending prospective clinical validation. These targets merit further investigation to elucidate their specific roles and therapeutic potential. Subsequent investigations should prioritize prospective validation in clinical cohorts and functional studies to establish causal mechanisms and assess therapeutic relevance.

## Supplementary Material

Suppl 1 Characteristics of the LC and VTE datasets included in this study.

Suppl 2 Gene sets used for the identification of shared crosstalk genes between lung cancer and venous thromboembolism.

Suppl 3 Preprocessing and normalization of the LC and VTE datasets.

Suppl 4 Identification of VTE-associated differentially expressed genes, WGCNA modules, and shared crosstalk genes.

Suppl 5 GSVA and immune infiltration analyses in the VTE cohort.

Suppl 6 Predicted transcriptional and posttranscriptional regulatory interactions involving the signature genes.

## Data Availability

The datasets analyzed during the current study are publicly available in the Gene Expression Omnibus (GEO) repository. The lung cancer datasets can be accessed via GSE30219 and GSE19188. The venous thromboembolism datasets are available under GSE48000 and GSE19151. All data generated or analyzed during this study are included in this published article and its Supplementary information files. The analysis code supporting the findings of this study is not publicly available due to institutional restrictions but can be made available from the corresponding author on reasonable request.
