# Supplementary material for: A Mitochondrial-Related Gene Signature for Diagnosis and Immune Microenvironment Modulation in Lung Cancer and Venous Thromboembolism
Source: World J Oncol. 2026 Sep 4;17(5):683–704. doi: 10.14740/wjon2815 (PMC13568737; doi:10.14740/wjon2815)
Supplement: Supplementary file 1 [file wjon-17-05-683-s003.docx]

**Suppl3. Preprocessing and normalization of the LC and VTE datasets.**

(A, B) Boxplots of expression distributions in GSE30219 before (A) and after (B) normalization. (C, D) Boxplots of expression distributions in GSE19188 before (C) and after (D) normalization. (E, F) Boxplots of expression distributions in GSE48000 before (E) and after (F) normalization. (G, H) Boxplots of expression distributions in GSE19151 before (G) and after (H) normalization. Pink indicates the VTE group orange indicates the LC group, and control group samples are shown in blue. LC, lung cancer; VTE, venous thromboembolism.
