# Supplementary material for: A Mitochondrial-Related Gene Signature for Diagnosis and Immune Microenvironment Modulation in Lung Cancer and Venous Thromboembolism
Source: World J Oncol. 2026 Sep 4;17(5):683–704. doi: 10.14740/wjon2815 (PMC13568737; doi:10.14740/wjon2815)
Supplement: Supplementary file 2 [file wjon-17-05-683-s004.docx]

**Suppl4. Identification of VTE-associated differentially expressed genes, WGCNA modules, and shared crosstalk genes.**

(A, B) Volcano plots of DEGs in GSE48000 (A) and GSE19151 (B), comparing VTE samples with controls. (C) Venn diagram showing the overlap of DEGs between GSE48000 and GSE19151. (D, E) Heatmaps of the top 10 upregulated and top 10 downregulated VTEDEGs in GSE48000 (D) and GSE19151 (E). In volcano plots, pink represents VTE samples and light blue represents controls. In heatmaps, red represents higher expression and blue represents lower expression. (F) Determination of soft-thresholding power. (Left) Scale-free topology fit index. (Right) Mean connectivity. (G) Hierarchical clustering dendrogram showing module assignment for genes with the top 60% variance. (H) Module clustering based on hierarchical clustering. The upper panel shows the gene dendrogram, and the lower panel shows module colors. (I) Heatmap of correlations between gene modules and clinical traits (VTE vs. control). Correlation strength is indicated by |*r*| values, where < 0.3 suggests minimal and 0.3–0.5 indicates modest correlations. Color indicates direction of correlation, with pink representing positive and gray-green representing negative correlations. (J) Venn diagram showing the overlap among LCDEGs, VTEDEGs, LC_WGCNA, VTE_WGCNA, and MRGs. Abbreviations: LC, lung cancer; VTE, venous thromboembolism; MRGs, mitochondria-related genes. DEGs, differentially expressed genes; VTEDEGs, venous thromboembolism–associated differentially expressed genes.
