# Supplementary material for: A Mitochondrial-Related Gene Signature for Diagnosis and Immune Microenvironment Modulation in Lung Cancer and Venous Thromboembolism
Source: World J Oncol. 2026 Sep 4;17(5):683–704. doi: 10.14740/wjon2815 (PMC13568737; doi:10.14740/wjon2815)
Supplement: Supplementary file 3 [file wjon-17-05-683-s005.docx]

**Suppl5. GSVA and immune infiltration analyses in the VTE cohort.**

(A) Heatmap of GSVA enrichment scores comparing VTE samples with controls. Red indicates higher pathway enrichment, and blue indicates lower enrichment. (B) Boxplots comparing pathway enrichment scores between VTE and control groups. (C) Boxplots assessing the relative quantities of immune cell subsets between control and VTE samples. (D) Heatmap showing correlations among immune cell populations in GSE19151. (E) Bubble plot showing correlations linking transcriptional activity with the extent of immune cell infiltration. Statistical significance is indicated as follows: ns, not significant (*p* ≥ 0.05); *, *p* < 0.05; **, *p* < 0.01; ***, *p* < 0.001. Correlation strength is based on |*r*|, where < 0.3 indicates negligible, 0.3–0.5 weak, and 0.5–0.8 moderate correlations. Color coding: light blue, control; pink, VTE. In panels B and C, red hues denote positive associations, whereas blue shades signify negative ones, with the saturation level corresponding to the magnitude of the correlation. Abbreviations: ssGSEA, single-sample gene set enrichment analysis; VTE, venous thromboembolism.
